# LncRNA MALAT1 gene polymorphisms in coronary artery disease: a case–control study in a Chinese population

**DOI:** 10.1042/BSR20182213

**Published:** 2019-03-19

**Authors:** Weina Hu, Hanxi Ding, An Ouyang, Xiaohong Zhang, Qian Xu, Yunan Han, Xueying Zhang, Yuanzhe Jin

**Affiliations:** 1 The Department of Cardiology, The Fourth Affiliated Hospital of China Medical University, Shenyang 110034, China; 2 The First Affiliated Hospital of China Medical University, and Key Laboratory of Cancer Etiology and Prevention (China Medical University), Liaoning Provincial Education Department, Shenyang 110001, China; 3 Department of Kinesiology and Health Promotion, University of Kentucky, Lexington, KY 40506, U.S.A.; 4 Division of Public Health Sciences, Department of Surgery, Washington University School of Medicine, St. Louis, MO 63110, U.S.A.

**Keywords:** Coronary artery disease, Long noncoding RNA, Single nucleotide polymorphism

## Abstract

**Background:** Coronary artery disease (CAD) is one of the main fatal diseases all over the world. CAD is a complex disease, which has multiple risk factors mechanisms. In recent years, genome-wide association study (GWAS) had revealed single nucleotide polymorphism genes (SNPs) which were closely related with CAD risks. The relationship between long non-coding RNA (lncRNA) MALAT1 (metastasis-associated lung adenocarcinoma transcript 1) and CAD risk is largely unknown. To our knowledge, this is the first study which demonstrated the interaction effects of SNP–SNP and SNP–environment with CAD risk. In general, our case–control study is to detect the association between MALAT1 (rs619586, rs4102217) SNPs and CAD risk. ***Methods:*** Three hundred and sixty-five CAD patients and three hundred and eighty-four matched control participants blood samples were collected in Liaoning province, China. Two polymorphisms (rs619586, rs4102217) in lncRNA MALAT1 were genotyped by KASP platform. ***Results:*** In a stratified analysis, we found that non-drinkers with GC genotype and the recessive model of rs4102217 had higher CAD risk (*P*=0.010, odds ratio (OR): 1.96, 95% confidence interval (CI) = 1.17–3.28; *P*=0.026, OR: 1.73, 95% CI = 1.07–2.79) and diabetes mellitus (DM) history group (*P*=0.010, OR: 4.07, 95% CI = 1.41–11.81; *P*=0.019, OR: 3.29, 95% CI = 1.22–8.88). In SNP–SNP interactions analysis between MALAT1 and CAD risk, we found rs4102217 had an increase in smokers (GG: OR: 2.04, 95% CI = 1.42–2.92; CC+GC: OR: 2.64, 95% CI = 1.64–4.26) and a decrease in drinkers (CC+GC: OR: 0.33, 95% CI = 0.20–0.55). Smokers with MALAT1 rs619586 AA genotype (OR: 2.20, 95% CI = 1.57–3.07) and GG+AG genotype (OR: 2.11, 95% CI = 1.17–3.81) had a higher risk of CAD. Moreover, drinkers with AA genotype (OR: 0.22, 95% CI = 0.10–0.48) and GG+AG genotype (OR: 0.38, 95% CI = 0.22–0.65) had a lower risk of CAD. According to the MDR software, MALAT1 rs4102217 polymorphism-smoking-drinking was the best interaction model, which has higher risk of CAD (Testing Bal.ACC. = 0.6979). **Conclusion:** Our study demonstrated that the GC genotype and the recessive model of rs4102217 potentially increased CAD risk in some specific group.

## Introduction

Currently, coronary artery disease (CAD) is one of the leading cause of deaths worldwide [1,2]. The 2017 China Reports of Cardiovascular Diseases showed that the prevalence of CAD disease in China is still on the rise [3]. In next decades, CAD is expected to cause approximately 3.4 million deaths in China.

Multiple risk factors contribute to CAD development [4,5]. Recently, genome-wide association study (GWAS) has revealed single nucleotide polymorphism genes (SNPs) which are related with CAD risk. As genetic inheritance is an inevitable risk factor in the development of CAD, it is critical to identify the SNP locus of CAD risk [4,6–8].

Long non-coding RNA (lncRNA) is one of the most important members of non-coding RNA family. Recently, numerous studies have reported that lncRNA plays a regulatory role in other complex diseases, such as cancer, ischemic stroke, Alzheimer’s disease, and heart disease [9–14].

Particularly, MALAT1 (metastasis-associated lung adenocarcinoma transcript 1) known as non-coding nuclear-enriched abundant transcript 2 (NEAT2) is the one of the first found lncRNA with widely expression in various mammalian species [14]. MALAT1 is located on chromosome 11q13.1, majorly expressed in nucleus and is highly conserved. Moreover, it has high expression in various human tissues [15–18].

Many studies have shown that lncRNA MALAT1 was associated with CAD risk [19]. In 2012, Zhuo et al. [19] demonstrated that rs619586A→G regulated the expression of XBP1, and ultimately prevented the proliferation and metastasis of pulmonary artery endothelial cells. Vausort et al. [40] found that MALAT1 levels in peripheral blood cells was significantly higher in acute myocardial infarction patients compared with controls. Wang et al. [20] found that MALAT1 SNP rs619586 AG/GG genotypes may protect against the occurrence of CAD, but not rs11227209, rs664589, and rs3200401. To our knowledge, no evidence demonstrates the relationship between MALAT1 SNP and CAD risk. In addition, we further conducted SNP–SNP and SNP–environmental factors interaction analysis [20].

In summary, we conducted a case–control study, analyzed statistical methods with clinical data, and detected relationship between MALAT1 (rs619586, rs4102217) SNPs and CAD risk. The aim of the present study was to identify predictive biomarkers for CAD risk and establish an experimental basis to improve understanding of the etiology and the mechanism of CAD.

## Materials and methods

### Patients

The Ethical Committee of the Fourth Affiliated Hospital of the China Medical University approved this research project and written informed consent was obtained. All clinical investigations have been conducted according to the principles described in the Declaration of Helsinki. A total of 749 participants were recruited in the present study, including 365 CAD patients and 384 matched controls. All diagnoses were made based on 2014 AHA/ACC guidelines for the management of NSTEACS and Third Universal Definition of Myocardial Infarction, with confirmation by coronary angiography [21]. Coronary artery and Gensini score assessed the severity of CAD [22,23]. A total of 384 gender and age frequency-matched controls were included from a health screening program from the community of the same area, Liaoning Province, China from 2012 to 2014. Peripheral venous blood specimens were collected from participants and stored at −20°C until use.

Exclusion criteria included history of malignancies, rheumatoid arthritis, and connective tissue diseases, organ transplantation, and long-term use of immunosuppressive medication.

### SNP selection and genotyping

Genetic polymorphisms were screened by HapMap database. Haploview 4.2 was used to select, and according to Chinese Beijing Han population (CHB), unbalanced R2 value more than 0.8, and the minimum allele frequency (Minor Allele Frequency, MAF) was greater than 5%. F-SNP software (http://compbio.cs.queensu.ca/F-SNP/) was used to predict the possible functions of these selected sites. At last, we selected MALAT1 tagSNPs according to the literature [24]. The most common SNPs on *MALAT1* gene were two sites (rs4102217, rs619586).

Genomic DNA was extracted using a previously published method and diluted to working concentrations of 20 ng.l^−1^ for genotyping. The assay was performed by Gene Company (Shanghai, China), using allele-specific PCR using KASPar (KASP) reagents (LGC Genomics, Hoddesdon, U.K.). For quality control, we repeatedly genotyped 10% of the total samples at one time. The concordance rate of these repeated samples reached 100%, which demonstrated that the genotyping results were reliable.

### Statistical analysis

Between-group differences of gender as well as the Hardy–Weinberg Equilibrium were compared by the χ^2^ test, and ANOVA was conducted for age variability. Multivariate logistic regression with adjustments for age and gender was used to show the association between selected gene polymorphisms with CAD risk. The haplotype of each gene was analyzed using SHEsis software [25,26]. All MALAT1 gene polymorphisms identified in the best models of gene–gene interactions were calculated using MDR software (version 3.0.2). The combined effect of selected SNP–SNP interactions in the best model was determined by multivariate logistic regression adjusted for age and gender. The association between gene polymorphisms and clinical parameters was performed by χ^2^ test; the differences for the clinical parameters amongst different polymorphism groups were compared by using *t* test. *P*-value <0.05 was considered significant.

## Results

### The baseline characteristics of the subjects

The demographic characteristics of CAD and control subjects were shown in Supplementary Table S1. There was no significant difference in the age (57.0 ± 8.1 compared with 57.4 ± 8.8 years) and gender (male 73.7% compared with female 75.6%) between the CAD and control groups. There were remarkable differences in the two groups of CAD risk factors, including smoking, drinking, hypertension, diabetes, cerebrovascular disease, total cholesterol, triglyceride, high-density lipoprotein, and low-density lipoprotein (*P*<0.05).

### The association of SNPs in *MALAT1* gene with CAD risk

We genotyped two polymorphisms of lncRNA MALAT1 gene (rs619586 and rs4102217) (Table 1). The two SNPs were conformed to the Hardy–Weinberg Equilibrium. However, we did not find any relationship between the two SNPs and CAD risk (*P*>0.05).

**Table 1:** The association of lncRNA MALAT1 polymorphisms and CAD risk^1^

SNPs	CON (%)	CAD (%)	CAD compared with CON	
			*P*^2^	OR (95% CI)
MALAT1 rs4102217				
GG	275 (77.9)	243 (72.1)		1 (Ref)
GC	78 (22.1)	94 (27.9)	0.076	1.37 (0.97–1.94)
CC	11 (3.8)	8 (3.2)	0.705	0.84 (0.33–2.12)
CC+GC compared with GG			0.120	1.30 (0.93–1.82)
CC compared with GC+GG			0.575	0.77 (0.31–1.93)
C compared with G			0.233	1.20 (0.89–1.60)
*P*_HWE_^2^	0.068			
MALAT1 rs619586				
AA	309 (87.0)	293 (85.4)		1 (Ref)
AG	46 (13.0)	50 (14.6)	0.531	1.15 (0.75–1.77)
GG	2 (0.6)	1 (0.3)	0.667	0.59 (0.05–6.56)
GG+AG compared with AA			0.589	1.13 (0.74–1.72)
GG compared with G+AA			0.628	0.55 (0.05–6.12)
G compared with A			0.671	1.09 (0.73–1.63)
*P*_HWE_^2^	0.839			

Abbreviations: CI, confidence interval; CON, control, OR, odds ratio; NCBI Ref, the reference frequencies of these polymorphisms in Beijing Han, China in NCBI database. ^1^Using logistic regression adjusted by sex and age. ^2^Means Hardy−Weinberg Equilibrium in population.

### The association of MALAT1 polymorphisms with CAD risk stratified by individual characteristics

To minimize other CAD risk factors influences, we carried out a stratified analysis (Table 2). The GC genotype and the recessive model of rs4102217 polymorphism showed stronger relations with higher CAD risk both in non-drinkers (*P*=0.010, odds ratio (OR): 1.96, 95% confidence interval (CI) = 1.17–3.28; *P*=0.026, OR: 1.73, 95% CI = 1.07–2.79, respectively) and in diabetes mellitus (DM) history group (*P*=0.010, OR: 4.07, 95% CI = 1.41–11.81; *P*=0.019, OR: 3.29, 95% CI = 1.22–8.88, respectively). We did not observe any changes in rs619586 (*P*>0.05).

**Table 2:** The association of lncRNA MALAT1 polymorphisms and CAD risk stratified by host characteristics

Variables	Genotype	CAD compared with CON	*P*^1^	OR (95% CI)
MALAT1 rs4102217				
Gender				
Male	GG	189/201		1 (Ref)
	GC	65/58	0.319	1.23 (0.82–1.84)
	CC	6/7	0.871	0.91 (0.30–2.76)
	CC+GC compared with GG		0.372	1.19 (0.81–1.76)
	CC compared with GC+GG		0.799	0.87 (0.29–2.61)
Female	GG	54/74		1 (Ref)
	GC	27/20	0.072	1.86 (0.95–3.68)
	CC	2/4	0.696	0.71 (0.12–4.03)
	CC+GC compared with GG		0.121	1.67 (0.87–3.18)
	CC compared with GC+GG		0.545	0.59 (0.11–3.30)
Age (years)				
≤60	GG	149/162		1 (Ref)
	GC	53/44	0.251	1.31 (0.83–2.07)
	CC	8/6	0.487	1.47 (0.50–4.37)
	CC+GC compared with GG		0.198	1.33 (0.86–2.06)
	CC compared with GC+GG		0.529	1.42 (0.48–4.18)
>60	GG	94/113		1 (Ref)
	GC	41/34	0.164	1.47 (0.86–2.53)
	CC	0/5	NA	NA
	CC+GC compared with GG		0.361	1.28 (0.76–2.17)
	CC compared with GC+GG		NA	NA
Smoking				
Ever smoker	GG	168/144		1 (Ref)
	GC	58/63	0.298	1.28 (0.80–2.04)
	CC	7/4	0.568	1.45 (0.41–5.11)
	CC+GC compared with GG		0.257	1.30 (0.83–2.03)
	CC compared with GC+GG		0.627	1.37 (0.39–4.80)
Never smoker	GG	75/131		1 (Ref)
	GC	36/39	0.080	1.62 (0.94–2.77)
	CC	1/7	0.185	0.24 (0.03–1.99)
	CC+GC compared with GG		0.205	1.40 (0.83–2.36)
	CC compared with GC+GG		0.146	0.21 (0.03–1.72)
Alcohol drinking				
Drinker	GG	65/151		1 (Ref)
	GC	24/50	0.780	1.09 (0.61–1.92)
	CC	2/5	0.909	1.11 (0.20–6.20)
	CC+GC compared with GG		0.787	1.08 (0.62–1.88)
	CC compared with GC+GG		0.986	1.02 (0.19–5.49)
Non-drinker	GG	178/124		1 (Ref)
	GC	70/28	**0.010**	**1.96 (1.17–3.28)**
	CC	6/6	0.557	0.70 (0.21–2.30)
	CC+GC compared with GG		**0.026**	**1.73 (1.07–2.79)**
	CC compared with GC+GG		0.396	0.60 (0.18–1.96)
HBP				
Yes	GG	135/89		1 (Ref)
	GC	54/23	0.168	1.48 (0.85–2.60)
	CC	4/2	0.945	1.07 (0.18–6.23)
	CC+GC compared with GG		0.173	1.46 (0.85–2.52)
	CC compared with GC+GG		0.941	1.07 (0.19–6.05)
NO	GG	107/186		1 (Ref)
	GC	40/55	0.383	1.24 (0.77–2.00)
	CC	4/9	0.647	0.75 (0.22–2.57)
	CC+GC compared with GG		0.498	1.17 (0.74–1.85)
	CC compared with GC+GG		0.611	0.73 (0.22–2.45)
DM				
Yes	GG	62/39		1 (Ref)
	GC	29/5	**0.010**	**4.07 (1.41–11.81)**
	CC	0/1	NA	NA
	CC+GC compared with GG		**0.019**	**3.29 (1.22-8.88)**
	CC compared with GC+GG		NA	NA
No	GG	181/235		1 (Ref)
	GC	65/73	0.412	1.18 (0.80–1.74)
	CC	8/10	0.884	1.07 (0.41–2.81)
	CC+GC compared with GG		0.426	1.16 (0.80–1.69)
	CC compared with GC+GG		0.963	1.02 (0.40–2.65)
LDL				
High	GG	44/56		1 (Ref)
	GC	14/16	0.787	1.12 (0.49–2.59)
	CC	2/2	0.801	1.29 (0.18–9.59)
	CC+GC compared with GG		0.732	1.15 (0.52–2.54)
	CC compared with GC+GG		0.819	1.26 (0.17–9.26)
Normal	GG	174/211		1 (Ref)
	GC	65/60	0.183	1.32 (0.88–1.98)
	CC	5/9	0.489	0.68 (0.22–2.06)
	CC+GC compared with GG		0.293	1.23 (0.83–1.82)
	CC compared with GC+GG		0.414	0.63 (0.21–1.91)
Low	GG	12/6		1 (Ref)
	GC	8/2	0.564	1.84 (0.23–14.68)
	CC	0/0	NA	NA
	CC+GC compared with GG		0.564	1.84 (0.23–14.68)
	CC compared with GC+GG		NA	NA
MALAT1 rs619586				
Gender				
Male	AA	225/223		1 (Ref)
	AG	33/36	0.699	0.91 (0.54–1.50)
	GG	1/1	0.923	1.15 (0.07–18.62)
	GG+AG compared with AA		0.709	0.91 (0.55–1.50)
	GG compared with AG+AA		0.956	1.08 (0.07-17.50)
Female	AA	68/86		1 (Ref)
	AG	17/10	0.069	2.19 (0.94–5.11)
	GG	0/1	NA	NA
	GG+AG compared with AA		0.103	1.99 (0.87–4.53)
	GG compared with AG+AA		NA	NA
Age (years)				
≤60	AA	175/184		1 (Ref)
	AG	29/25	0.487	1.23 (0.69–2.18)
	GG	1/1	0.993	1.01 (0.06–16.35)
	GG+AG compared with AA		0.493	1.22 (0.69–2.15)
	GG compared with AG+AA		0.990	1.02 (0.06–16.40)
>60	AA	118/125		1 (Ref)
	AG	21/21	0.898	1.04 (0.54–2.01)
	GG	0/1	NA	NA
	GG+AG compared withAA		0.995	1.00 (0.52–1.91)
	GG compared with AG+AA		NA	NA
Smoking				
Ever smoker	AA	205/159		1 (Ref)
	AG	30/24	0.983	0.99 (0.56–1.77)
	GG	1/1	0.949	0.91 (0.06–14.86)
	GG+AG compared with AA		0.970	0.99 (0.56–1.75)
	GG compared with AG+AA		0.928	0.88 (0.05–14.28)
Never smoker	AA	88/150		1 (Ref)
	AG	20/22	0.186	1.57 (0.81–3.05)
	GG	0/1	NA	NA
	GG+AG compared with AA		0.235	1.49 (0.77–2.89)
	GG compared with AG+AA		NA	NA
Alcohol drinking				
Drinker	AA	78/174		1 (Ref)
	AG	13/26	0.800	1.10 (0.53–2.26)
	GG	0/1	NA	NA
	GG+AG compared with AA		0.884	1.06 (0.52–2.16)
	GG compared with AG+AA		NA	NA
Non-drinker	AA	215/135		1 (Ref)
	AG	37/20	0.481	1.24 (0.68–2.27)
	GG	1/1	0.834	0.74 (0.04–12.70)
	GG+AG compared with AA		0.515	1.22 (0.67–2.20)
	GG compared with AG+AA		0.800	0.69 (0.04–11.75)
HBP				
Yes	AA	165/90		1 (Ref)
	AG	31/16	0.957	1.02 (0.53–1.98)
	GG	0/2	NA	NA
	GG+AG compared with AA		0.758	0.90 (0.48–1.72)
	GG compared with AG+AA		NA	NA
No	AA	127/219		1 (Ref)
	AG	19/30	0.814	1.08 (0.58–2.01)
	GG	1/0	NA	NA
	GG+AG compared with AA		0.695	1.13 (0.61–2.09)
	GG compared with AG+AA		NA	NA
DM				
Yes	AA	71/39		1 (Ref)
	AG	17/7	0.778	1.15 (0.43–3.12)
	GG	0/0	NA	NA
	GG+AG compared with AA		0.778	1.15 (0.43–3.12)
	GG compared with AG+AA		NA	NA
No	AA	222/269		1 (Ref)
	AG	33/39	0.997	1.00 (0.61–1.65)
	GG	1/2	0.802	0.73 (0.07–8.22)
	GG+AG compared with AA		0.959	0.99 (0.60–1.61)
	GG compared with AG+AA		0.768	0.70 (0.06–7.78)
LDL				
High	AA	56/54		
	AG	6/15	0.065	0.38 (0.14–1.06)
	GG	1/1	0.936	0.89 (0.05–14.86)
	GG+AG compared with AA		0.072	0.41 (0.16–1.08)
	GG compared with AG+AA		1.000	1.00 (0.06–16.55)
Normal	AA	204/246		
	AG	36/29	0.128	1.50 (0.89–2.54)
	GG	0/1	NA	NA
	GG+AG compared with AA		0.159	1.46 (0.86–2.45)
	GG compared with AG+AA		NA	NA
Low	AA	16/6		
	AG	4/2	0.782	0.74 (0.09–8.37)
	GG	0/0	NA	NA
	GG+AG compared with AA		0.782	0.74 (0.09–6.37)
	GG compared with AG+AA		NA	NA

Abbreviations: CON, control; HBP, high blood pressure; LDL, low-density lipoprotein. ^1^Using Logistic Regression adjusted by gender and age.

Values in bold represent statistical significance.

### The association between haplotype of MALAT1 SNPs and CAD risk

Haplotypes with a frequency less than 0.03 would be excluded from our analysis (Table 3). There were no significant differences in the haplotype analysis (*P*>0.05).

**Table 3:** The association of haplotype of *MALAT1* gene and CAD risk

Haplotype	Case (%)	Control (%)	*P*	OR (95% CI)
CA	88.83 (0.135)	97.97 (0.141)	0.838	0.97 (0.71–1.32)
GA	520.17 (0.788)	549.03 (0.789)	0.560	1.08 (0.83–1.41)
GG	39.83 (0.060)	48.97 (0.070)	0.508	0.86 (0.56–1.33)

Used SHEsis software for analysis (http://analysis.bio-x.cn/).

### Multidimensional analysis of SNP–SNP interactions between MALAT1 and CAD risk

To investigate SNP–SNP interactions between MALAT1 and CAD risk, multiple logistic regression analysis was employed (Table 4). We found rs4102217 had interactions with smokers (GG: OR: 2.04, 95% CI = 1.42–2.92; CC+GC: OR: 2.64, 95% CI = 1.64–4.26) and drinkers (CC+GC: OR: 0.33, 95% CI = 0.20–0.55). We also found MALAT1 rs619586 AA genotype (OR: 2.20, 95% CI = 1.57–3.07) and GG+AG genotype (OR: 2.11, 95% CI = 1.17–3.81) had higher risks of CAD in smokers. In addition, rs619586 AA genotype (OR: 0.22, 95% CI = 0.10–0.48) and GG+AG genotype (OR: 0.38, 95% CI = 0.22–0.65) had lower risks of CAD in drinkers. Meanwhile, MDR software was used to further investigate the locus–locus interactions of MALAT1 and CAD risk (Table 5). We found that the three factors model, MALAT1 rs4102217 polymorphism-smoking-drinking was the best interaction model. The maximum testing accuracy was 0.6979, the maximum CV consistency was 10/10. Furthermore, we conducted cumulative effect of the interacting factors of MALAT1 SNPs on CAD risk (Table 6). MALAT1 rs4102217 polymorphism-smoking-drinking was considered as an integrated risk factor. CAD patients were divided into five groups of risks: group 1:0 risk genotype; group 2: 1 risk genotype; group 3: 2 risk genotypes; group 4: 3 risk genotypes; and group 5: 4 risk genotypes. After adjustment of gender and age, group 2 had a higher risk of CAD (*P*=0.009, OR: 1.84, 95% CI = 1.17–2.90).

**Table 4:** The interaction of three MALAT1 polymorphisms with environmental factors in CAD risk

		Smoking		Drinking	
		Never Smoker	Ever Smoker	Non-drinker	Drinker
MALAT1 rs4102217					
GG	Case/control	75/131	168/144	178/124	65/151
	OR (95% CI)	1 (Ref)	2.04 (1.42–2.92)	1 (Ref)	0.30 (0.21–-0.43)
CC+GC	Case/control	37/46	65/43	76/34	26/55
	OR (95% CI)	1.41 (0.84–2.36)	2.64 (1.64–4.26)	1.56 (0.98–2.48)	0.33 (0.20–0.55)
		*P*_interaction_=0.825		*P*_interaction_=0.207	
		OR = 0.93, 95% CI = 0.47–1.84		OR = 0.624, 95% CI = 0.30–1.30	
MALAT1 rs619586					
AA	Case/control	88/150	205/19	215/135	78/174
	OR (95% CI)	1 (Ref)	2.20 (1.57–3.07)	1 (Ref)	0.22 (0.10–0.48)
GG+AG	Case/control	20/23	31/25	38/21	13/27
	OR (95% CI)	1.48 (0.77–2.85)	2.11 (1.17–3.81)	1.41 (0.84–2.37)	0.38 (0.22–0.65)
		*P*_interaction_=0.355		*P*_interaction_=0.753	
		OR = 0.66, 95% CI = 0.28–1.58		OR = 0.86, 95% CI = 0.34–2.18	

*P* for interaction used logistic regession adjusted by gender, age.

**Table 5:** Gene–gene interaction models for MALAT1 two polymorphisms for CAD risk by MDR analysis

Model	Training Bal. Acc.	Testing Bal. Acc.	Sign Test (*P*)	CV Consistency	*P* for permutation test
Drinking	0.6598	0.6601	10 (0.0010)	10/10	0.0000–0.0010
Smoking-Drinking	0.6790	0.6789	10 (0.0010)	10/10	0.0000–0.0010
MALAT1 rs4102217-smoking-drinking^1^	0.6995	0.6979	10 (0.0010)	10/10	0.0000–0.0010
MALAT1 rs4102217-MALAT1 rs619586-smoking-drinking	0.7054	0.6900	10 (0.0010)	10/10	0.0000–0.0010

The best model was selected as the one with the maximum testing accuracy and maximum CV consistency. ^1^In this study, the best interaction model was the three-factor model including MALAT1 rs4102217 polymorphism-smoking-drinking.

**Table 6:** Cumulative effect of the interacting factors of MALAT1 SNPs on CAD

Number of interacting genotypes	Total population		
	Cases/controls	*P*^1^ value	OR (95% CI)
MALAT1 rs41022173-rsMALAT1 rs619568-smoking-drinking			
0	56/75		1 (Ref)
1	134/92	**0.009**	**1.84 (1.17–2.90)**
2	105/127	0.904	0.97 (0.60–1.58)
3	31/51	0.266	0.70 (0.38–1.31)
4	4/3	0.610	1.50 (0.32–7.13)
	*P*_trend_=0.198		

^1^, Adjusted by sex and age.

Values in bold represent statistical significance.

### The association between MALAT1 polymorphisms and clinical parameters

To analyze the relationship between clinical parameters and genetic polymorphisms, the main genetic polymorphisms model was selected. In general, if the *P*-value of the dominant model was less than the recessive model, then the dominant model was selected, otherwise the recessive model was selected (Table 7). In our data, the dominant gene model was selected both in MALAT1 rs4102217 and in MALAT1 rs619586. We found that MALAT1 rs4102217 CC+GC genotype was higher in uric acid in both qualitative analysis (*P*=0.014) and quantitative analysis (359.35 ± 109.90 compared with 327.06 ± 115.38 μmol/l; *P*=0.015). Moreover, we found that the wild-type triglyceride for MALAT1 rs619586 was lower than the mutation (*P*=0.003), and the content was significantly lower (1.82 ± 1.39 compared with 3.12 ± 3.58 mmol/l; *P*=0.017). High-density lipoprotein in wild-type is significantly higher than mutation-type for MALAT1 rs619586 (1.02 ± 0.29 compared with 0.92 ± 0.24 mmol/l, *P*=0.032). There was a dramatic increase in uric acid in the wild-type than in the mutation-type (342.75 ± 101.42 compared with 385.04 ± 159.87 μmol/l, *P*=0.013). In addition, we analyzed the association of MALAT1 SNPs with severity of coronary artery by analyzing numbers of coronary artery lesion branches and Gensini score. But there was no statistical significance (*P*>0.05).

**Table 7:** The association of MALAT1 SNPs and clinical features

Variation	Wild-type	Mutated-type	Wild-type	Mutated-type	*P*
**MALAT1 rs4102217**					
Smoking	*P*=0.327				
No	75 (30.9)	37 (36.3)	/	/	/
Yes	168 (69.1)	65 (63.7)	/	/	/
Drinking	*P*=0.809				
No	178 (73.3)	76 (74.5)	/	/	/
Yes	65 (26.7)	26 (25.5)	/	/	/
HBP	*P*=0.854				
No	107 (44.2)	44 (43.1)	/	/	/
Yes	135 (55.8)	58 (56.9)	/	/	/
Diabetes	*P*=0.575				
No	181 (74.5)	73 (71.6)	/	/	/
Yes	62 (25.5)	29 (28.4)	/	/	/
Cerebrovascular disease	*P*=0.570				
No	208 (86.0)	90 (88.2)	/	/	/
Yes	34 (14.0)	12 (11.8)	/	/	/
Hyperlipidemia	*P*=0.521				
No	114 (46.9)	44 (43.1)	/	/	/
Yes	129 (53.1)	58 (56.9)	/	/	/
Blood Glucose	*P*=0.747		8.20 ± 4.14	8.53 ± 5.10	0.538
Normal	80 (33.8)	32 (31.7)			
High	156 (65.8)	69 (68.3)			
Low	1 (0.4)	0 (0)			
Total cholesterol	*P*=0.719		4.43 ± 1.19	4.55 ± 1.21	0.385
Normal	178 (77.4)	71 (75.5)			
High	52 (22.6)	23 (24.5)			
Triacylglyceride	*P*=0.373		1.90 ± 1.58	2.29 ± 2.58	0.168
Normal	193 (83.9)	75 (79.8)			
High	37 (16.1)	19 (20.2)			
High-density lipoprotein	*P*=0.846		0.99 ± 0.27	0.98 ± 0.29	0.708
Normal	105 (45.7)	46 (48.9)			
High	2 (0.9)	1 (1.1)			
Low	123 (53.5)	47 (50.0)			
Low-density lipoprotein	*P*=0.510		2.94 ± 1.05	2.91 ± 0.84	0.795
Normal	174 (75.7)	70 (74.5)			
High	44 (19.1)	16 (17.0)			
Low	12 (5.2)	8 (8.5)			
Urea nitrogen	*P*=0.980		6.27 ± 3.47	6.44 ± 5.18	0.732
Normal	213 (89.1)	91 (89.2)			
High	26 (10.9)	11 (10.8)			
Creatinine	*P*=0.497		89.93 ± 50.92	88.80 ± 31.66	0.835
Normal	227 (95.0)	95 (93.1)			
High	12 (5.0)	7 (6.9)			
Uric acid	***P*=0.014**		359.35 ± 109.90	327.06 ± 115.38	**0.015**
Normal	190 (79.2)	92 (90.2)			
High	50 (20.8)	10 (9.8)			
Coronary artery lesions	*P*=0.307				
One	57 (27.9)	19 (21.3)	/	/	/
Two	39 (19.1)	23 (25.8)	/	/	/
Three or more	108 (52.9)	47 (52.8)	/	/	/
Gensini score			54.30 ± 36.03	50.98 ± 31.89	0.458
**MALAT1 rs619586**					
Smoking	*P*=0.192				
No	88 (30.0)	20 (39.2)	/	/	/
Yes	205 (70.0)	31 (60.8)	/	/	/
Drinking	*P*=0.866				
No	215 (73.4)	38 (74.5)	/	/	/
Yes	78 (26.6)	13 (25.5)	/	/	/
HBP	*P*=0.569				
No	127 (43.5)	20 (39.2)	/	/	/
Yes	165 (56.5)	31 (60.8)	/	/	/
Diabetes	*P*=0.169				
No	222 (75.8)	34 (66.7)	/	/	/
Yes	71 (24.2)	17 (33.3)	/	/	/
Cerebrovascular disease	*P*=0.077				
No	256 (87.7)	40 (78.4)	/	/	/
Yes	36 (12.3)	11 (21.6)	/	/	/
Hyperlipidemia	*P*=0.434				
No	138 (47.1)	21 (41.2)	/	/	/
Yes	155 (52.9)	30 (58.8)	/	/	/
Blood glucose	*P*=0.169		8.06 ± 3.80	9.10 ± 6.60	0.282
Normal	90 (31.5)	20 (40.0)			
High	195 (68.2)	29 (58.0)			
Low	1 (0.3)	1 (2.0)			
Total cholesterol	*P*=0.619		4.49 ± 1.14	4.49 ± 1.56	0.965
Normal	208 (75.4)	37 (78.7)			
High	68 (24.6)	10 (21.3)			
Triacylglyceride	***P*=0.003**		1.82 ± 1.39	3.12 ± 3.58	**0.017**
Normal	236 (85.5)	32 (68.1)			
High	40 (14.5)	15 (31.9)			
High-density lipoprotein	*P*=0.150		1.02 ± 0.29	0.92 ± 0.24	**0.032**
Normal	136 (49.3)	17 (36.2)			
High	4 (1.4)	0 (0)			
Low	136 (49.3)	30 (63.8)			
Low-density lipoprotein	*P*=0.572		2.97 ± 1.00	2.75 ± 1.08	0.159
Normal	204 (73.9)	36 (76.6)			
High	56 (20.3)	7 (14.9)			
Low	16 (5.8)	4 (8.5)			
Urea nitrogen	*P*=0.077		6.22 ± 3.68	6.84 ± 5.81	0.316
Normal	261 (90.6)	42 (82.4)			
High	27 (9.4)	9 (17.6)			
Creatinine	*P*=0.451		90.11 ± 48.60	87.03 ± 29.51	0.662
Normal	273 (94.8)	47 (92.2)			
High	15 (5.2)	4 (7.8)			
Uric acid	*P*=0.952		342.75 ± 101.42	385.04 ± 159.87	**0.013**
Normal	239 (82.7)	42 (82.4)			
High	50 (17.3)	9 (17.6)			
Coronary artery lesions	*P*=0.535				
One	66 (26.7)	14 (31.1)	/	/	/
Two	49 (19.8)	11 (24.4)	/	/	/
Three or more	132 (53.4)	20 (44.4)	/	/	/
Gensini score			52.54 ± 33.26	54.48 ± 39.14	0.727

## Discussion

In our research, we found the GC genotype and the recessive model of rs4102217 polymorphism showed stronger relations with higher CAD risk both in non-drinkers and in DM history groups. In SNP–SNP interactions analysis between MALAT1 and CAD risk, MALAT1 rs4102217 polymorphism-smoking-drinking had a higher CAD risk. We also found that uric acid was higher in MALAT1 rs4102217 CC+GC genotype. Moreover, the wild-type of triacylglyceride for MALAT1 rs619586 was lower than the mutation-type. There were dramatic increases in uric acid and HDL in the wild-type than in the mutation-type.

MALAT1 is located on chromosome 11q13.1, and its length is 8.1 kb. MALAT-1 is a real non-coding RNA. Due to the lack of enough ORF and the location of its nucleus, the lncRNA cannot encode protein. In recent years, association between lncRNA, MALAT1, and cardiovascular diseases are popular [20,27–29]. Previous study showed that MALAT1 expression in atherosclerotic plaques was down-regulated and negatively related to age when compared with non-atherosclerotic artery specimens from CAD patients [30]. Another research found that peripheral matrix rather than the cell origin in CAD determined the classification of arterial and coronary vascular smooth muscle. The peripheral matrix lncRNA MALAT1 was sensitive in the peripheral matrix and can regulate the proliferation and migration of arterial and coronary vascular smooth muscle [31]. Thus, above evidences suggested that MALAT1 might be closely related to the development of CAD.

Rs4102217 is a variant of G/C in the exon region of *MALAT1* gene, which has not been reported yet. In our data, we found that there was no relationship in main effect analysis. However, in stratified analysis, the GC genotype and the recessive model of rs4102217 polymorphism showed stronger relations with higher CAD risk both in non-drinkers and in DM history groups. It could be used as a genetic locus to predict CAD risk. Rs619586 is the mutation of nucleotides A/G in the promoter region. Zhou et al. found that MALAT1 rs619586A/G is closely related to pulmonary hypertension risk [19]. Compared with the A loci causing PAH, the G genotype carrier has a lower risk. Another study pointed out that the rs619586 A/G mutation can directly up-regulate the expression of XBP1, and ultimately prevent the proliferation and metastasis of vascular endothelial cells [19]. Report from Wang et al. [20] suggested that rs619586 AG/GG genotypes and G allele were associated with a reduced risk of CAD. Li et al. [28] demonstrated that the functional MALAT1 polymorphism rs619586 A/G was significantly associated with CHD susceptibility in Chinese population. However, we did not find any association with CAD risk in rs619586 in main effect analysis. While, we obtained significant results in further interaction analysis of SNP–SNP and SNP–environment.

CAD is a complex disease involving multiple genes, multiple factors, such as age, sex, smoking, drinking, blood lipids, diabetes, and hypertension [32]. The development of CAD can not only be explained by SNPs. We conducted logistic regression analysis and MDR software analysis respectively to investigate the association between the SNP–SNP or SNP–environment interaction effects of MALAT1 and CAD risk [24,31,33,34]. Our data indicated that MALAT1 rs4102217 interacted with smokers and drinkers. MALAT1 rs619586 AA genotype and GG+AG genotype showed an elevated risk of CAD in smokers. AA genotype and GG+AG genotype showed a reduced risk of CAD in drinkers. To further investigate the relationship, MDR software was used to calculate the best prediction model and the prediction error of the training samples was measured by the test sample (the rest of the sample), while the evaluation for the size of the cross-validation consistency was used. We found that the three factors model, MALAT1 rs4102217 polymorphism-smoking-drinking was the most predictive model for the CAD risk, which had the maximum test accuracy and the maximum cross-validation consistency amongst the analysis results. They indicated that SNP–environment interaction effects were better to predict CAD than SNP alone.

In our research, we also analyzed the relationship between the polymorphism and clinical features. MALAT1 rs4102217 wild-type had more likely to suffer higher uric acid both qualitatively and quantitatively. For MALAT1 rs619586, we found the wild-type genotype carriers had more likely in high triglyceride and low high-density lipoprotein. Moreover, we used the numbers of coronary artery and Gensini score to assess coronary severity in our study. However, we did not find any differences.

Our research indicated that MALAT1 may be associated with the incidence of CAD, but currently, the mechanism of MALAT1 in CAD risk is not yet clear. MALAT1 probably performs its functions in two ways: alternative splicing or gene transcription regulation [35–37]. Lamond and Spector [38] found that MALAT-1 regulates the expression of post-transcriptional genes by regulating the distribution of SR (serine/arginine-rich) proteins which are rich in the nuclear spots. Moreover, MALAT-1 regulates the pre-mRNA (precursor messenger) level of SR protein. Weiner et al. [39] found that MALAT-1 alternatively regulates splicing by SR protein, including SRSF1, SRSF2, and SRSF3. Knocking down MALAT-1 will lead to ectopic of various splicing factors such as SF1, U2AF65, and SF3a60 [39]. Overexpression of SRSF1 results in alternative splicing results, which is similar to those obtained by knocking down MALAT1.

In summary, our study demonstrated that the polymorphisms (rs4102217, rs619586) of MALAT1 were associated with the CAD risk in Chinese population, which might predict CAD risk in the future.

## Limitations

Several limitations remained in our study: first, the sample size was relatively not sufficiently large in our study. The populations selected in our research were all Han people in Liaoning province. So the results of our study need to be validated in larger samples, other regions, and ethnic groups. Second, we only selected two sites of MALAT1. We need to test more sites to verify the association of CAD and MALAT1.

## Supporting information

**Supplenmentary Table S1:** The baseline of the subjects

## Abbreviations

CAD: coronary artery disease
CI: confidence interval
DM: diabetes mellitus
HDL: high density lipoprotein
lncRNA: long non-coding RNA
MALAT1: metastasis-associated lung adenocarcinoma transcript 1
NSTEACS: non ST elevation acute coronary syndrome
OR: odds ratio
PAH: pulmonary arterial hypertension
SNP: single nucleotide polymorphism
SR: serine/arginine-rich
