# Supplementary material for: LncRNA MALAT1 gene polymorphisms in coronary artery disease: a case–control study in a Chinese population
Source: Biosci Rep. 2019 Mar 19;39(3):BSR20182213. doi: 10.1042/BSR20182213 (PMC6422883; doi:10.1042/BSR20182213)
Supplement: Supplementary file 1 [file bsr-39-bsr20182213_Supp1.pdf]

LncRNA MALAT1 gene polymorphisms in coronary artery disease: a case-control study in a Chinese population

Weina Hu[1], Hanxi Ding[2], An Ouyang [3], Xiaohong Zhang[1], Qian Xu[2], Yunan Han[4], Xueying Zhang[1], Yuanzhe Jin[1,*]

[1]The Department of Cardiology, the Fourth Affiliated Hospital of China Medical University, Shenyang 110034, China

[2]The First Affiliated Hospital of China Medical University, and Key Laboratory of Cancer Etiology and Prevention (China Medical University), Liaoning Provincial Education Department, Shenyang 110001, China

[3]Department of Kinesiology and Health Promotion, University of Kentucky, Lexington, KY, U.S

[4]Division of Public Health Sciences, Department of Surgery, Washington University School of Medicine, St Louis, MO, 63110, U.S

***Corresponding author:**

Dr. Yuan-zhe Jin, MD/PhD, Professor

Vice-dean of the Fourth Affiliated Hospital of China Medical University

Director of the Department of Cardiology the Fourth Affiliated Hospital of China Medical University

4#East Chongshan Road,Huanggu District,Shenyang,Liaoning, Province, China.

Zip code: 110032

Tel: +86-18900916111

fax: +86-24-62042989

E-mail address: yzjin@cmu.edu.cn

Supplenmentary Table 1 The baseline of the subjects

Variables		CON vs.CAS		
		CON(%)	CAD(%)	*P* values
		**n=384**	**n=365**	
Gender				*P*=0.546
	Male	283(73.7)	276(75.6)	
	Female	101(26.3)	89(24.4)	
Age				*P*=0.986
	Mean±SD	57.0±8.1	57.4±8.8	
	Median	28/Feb/00	28/Feb/00	
	Range	35-79	29-81	
Smoking				***P*<0.001**
	Ever Smoker	198(51.6)	249(68.2)	
	Never Smoker	186(48.4)	116(31.8)	
Drinking				***P*<0.001**
	Drinker	218(56.8)	97(26.6)	
	Nondrinker	166(43.2)	268(73.4)	
HBP				***P*<0.001**
	Had	115(29.9)	209(57.4)	
	No	269(70.1)	155(42.6)	
Diabetes				***P*<0.001**
	Had	48(12.5)	96(26.3)	
	No	335(87.5)	269(73.7)	
Cerebrovascular Disease				***P*<0.001**
	Had	17(4.4)	50(13.7)	
	No	367(95.6)	314(86.3)	
Hyperlipidemia				***P*<0.001**
	Had	60(15.6)	196(53.7)	
	No	324(84.4)	169(46.3)	
Blood Glucose				***P*<0.001**
	High	106(27.8)	237(66.4)	
	Normal	270(70.9)	117(32.8)	
	Low	5(1.3)	3(0.8)	
Total Cholesterol				***P*<0.001**
	High	192(50.0)	80(23.3)	
	Normal	192(50.0)	263(76.7)	
Triglyceride				*P*=0.470
	High	58(15.2)	59(17.2)	
	Normal	323(84.8)	284(82.8)	
High-density Lipoprotein				***P*<0.001**
	High	12(3.1)	4(1.2)	
	Normal	320(84.0)	164(47.8)	
	Low	49(12.9)	175(51.0)	

Low-density Lipoprotein			***P*=0.021**
High	78(20.5)	64(18.7)	
Normal	295(77.4)	258(75.2)	
Low	8(2.1)	21(6.1)	

Note: CON:control; CAD:coronary artey disease. Smoking: smoking more than 1 cigarette per day and more than 6 months. Drinking: ethanol consumption in males (>140g/week), female (70g/week) and more than 1 year.
